# Biographical

**Published:** 1889-04

**Authors:** 


### BIOGRAPHICAL.

### The Late Dr. Crofoot, of Hartford, Conn.

HE HAS HONORED HIS CALLING.

When a man has faithfully and honestly followed his calling for a period of fifty years, and has laid down his armor with brightened surface, it is well to say commendable words of him. To Dr. Crofoot, who has rested from his labors, is due much praise for the honorable career with which he has adorned his profession. He turned his attention to his calling at a time when only mechanical aptitude was considered the prime requisite, and in this line he has gained a good name. No man in New England during his time has proved superior to him in the construction of artificial teeth, which were sought for far and near. It was not in the nature of Dr. Crofoot to degrade his calling by catering to cheap work. His first satisfaction was sought in the quality, rather than the price. Beginning practice as he did, before artificial teeth had become so large a factor in commerce, he found it very difficult to satisfy his tastes in teeth that in any sense corresponded to nature (for the earlier products were more like white beans than teeth), so he turned his personal attention to this work (for the trade) producing something better, and for a time finding himself successful, he attempted the larger manufacture, and joined his brother in Boston (until the brother's death). Dr. Crofoot received his first instructions in the office of Dr. Pratt in this city, in the days of Drs. Greenleaf and Crane. He commenced practice in Middletown in 1827, but the major portion of his practice has been in Hartford. His early dental associations in this city were among such honored ones as Drs. Wells, Riggs, Parmale and Preston, all of whom have left their calling richer in good works than they found it. Such a course is worthy the emulation of all.

Dr. Crofoot was inventive, and received a liberal compensation from the S. S. White Company in Philadelphia, who recognized the value of his invention. This was for an improvement in the head of the pin that is a part of the porcelain tooth manufactured by this company by the millions for the general trade. For superior skill in his line, Dr. Crofoot received many years ago an honorary degree from the Baltimore Dental College. He was held in esteem by all, and widely known by the older members of his profession.

For the genial, good-natured doctor we can all say: "Well done. You have earned the rest for your many years of labor, and your good works remain.

A PRACTITIONER.

